# Fentanyl-driven acceleration of racial, gender and geographical disparities in drug overdose deaths in the United States

**DOI:** 10.1371/journal.pgph.0000769

**Published:** 2023-03-22

**Authors:** Maria R. D’Orsogna, Lucas Böttcher, Tom Chou

**Affiliations:** 1 Department of Computational Medicine, University of California at Los Angeles, Los Angeles, California, United States of America; 2 Department of Mathematics, California State University at Northridge, Los Angeles, California, United States of America; 3 Department of Computational Science and Philosophy, Frankfurt School of Finance and Management, Frankfurt am Main, Germany; Université de Sherbrooke: Universite de Sherbrooke, CANADA

## Abstract

We examine trends in drug overdose deaths by race, gender, and geography in the United States during the period 2013–2020. Race and gender specific crude rates were extracted from the final National Vital Statistics System multiple cause-of-death mortality files for several jurisdictions and used to calculate the male-to-female ratios of crude rates between 2013 and 2020. We established 2013–2019 temporal trends for four major drug types: psychostimulants with addiction potential (T43.6, such as methamphetamines); heroin (T40.1); natural and semi-synthetic opioids (T40.2, such as those contained in prescription pain-killers); synthetic opioids (T40.4, such as fentanyl and its derivatives) through a quadratic regression and determined whether changes in the pandemic year 2020 were statistically significant. We also identified which race, gender and states were most impacted by drug overdose deaths. Nationwide, the year 2020 saw statistically significant increases in overdose deaths from all drug categories except heroin, surpassing predictions based on 2013–2019 trends. Crude rates for Black individuals of both genders surpassed those for White individuals for fentanyl and psychostimulants in 2018, creating a gap that widened through 2020. In some regions, mortality among White persons decreased while overdose deaths for Black persons kept rising. The largest 2020 mortality statistic is for Black males in the District of Columbia, with a record 134 overdose deaths per 100,000 due to fentanyl, 9.4 times more than the fatality rate among White males. Male overdose crude rates in 2020 remain larger than those of females for all drug categories except in Idaho, Utah and Arkansas where crude rates of overdose deaths by natural and semisynthetic opioids for females exceeded those of males. Drug prevention, mitigation and no-harm strategies should include racial, geographical and gender-specific efforts, to better identify and serve at-risk groups.

## Introduction

According to the Centers for Disease Control and Prevention (CDC) and the National Vital Statistics System (NVSS) 91,798 people died from injury or poisoning from drugs of abuse (opioids and psychostimulants) in 2020, corresponding to a 32% rise over 2019. Provisional mortality rates for 2021 and 2022 point to further rises in fatal overdoses [1]. The media often reports on these increases, as well as on rising non-fatal drug overdoses, or requests for drug-related emergency medical services starting in 2020 [2].

The recent surge in overdose deaths has been attributed to the many mental health stressors and uncertainties associated with COVID-19, and to pandemic-related disruptions to the drug supply that resulted in higher availability of lethal, illicit synthetic opioids such as fentanyl [3, 4]. However, drug abuse and age-adjusted overdose mortality rates have been steadily increasing over the past 20 years. In particular, the year 2013 is considered the beginning of a “third wave” of overdose fatalities [5, 6] marked by a shift from deaths due to prescription opioids towards deaths from synthetic opioids, primarily illicit fentanyl. Recent years have also seen more efforts to better understand differences among socio-economic, racial and gender groups in how, when and why drugs are first sought, how they are consumed, and what no-harm options might be the most effective [7–9]. However, few studies have provided a combined, in-depth analysis of gender, geographical and racial differences in drug-induced deaths, even prior to the pandemic [10]. For example, recent reports show that in 2020 the nationwide drug-induced crude rate for Black individuals surpassed that of White individuals for the first time since 1999 [11]. While interesting for its many societal implications, this analysis does not differentiate by drug type, gender or geography, hindering localized and/or specific intervention efforts. We aim to fill this gap by investigating mortality patterns during the third wave of drug overdoses in the United States, from 2013 to 2020. We focus on four major classes of drugs and verify whether the values recorded for the pandemic year 2020 are statistically significant compared to years prior.

## Methods

Overdose death rates for all years between 2013 and 2020 were obtained from the CDC WONDER database which tallies mortality, cause of death, and other relevant demographic data in the United States [12]. Overdose deaths were identified as drug poisoning as listed by the International Classification of Diseases, 10th Revision (ICD-10) using codes X40-X44 (unintentional), X60-X64 (suicide), X85 (homicide), or Y10-Y14 (undetermined intent). We do not distinguish among these categories and include all above manners of death in our evaluations. The type of drugs involved are listed via additional cause of death codes. We focus on the T40 class (poisoning by narcotics and psychodysleptics) which includes the relevant subclasses T40.1 (heroin), T40.2 (natural and semisynthetic opioids such as morphine and oxycodone, typically found in prescription pain-killers) and T40.4 (synthetic opioids other than methadone, such as fentanyl). Another relevant subclass is overdoses by psychostimulants with abuse potential (T43.6), a category which is listed under class T43 (poisoning by psychotropic drugs). Included in T43.6 are methamphetamines, amphetamines and 3,4-methylenedioxymethamphetamine (MDMA, ecstasy). For simplicity we will refer to the four categories as heroin (T40.1), prescription opioids (T40.2), fentanyl (T40.4) and methamphetamines (T43.6). We do not consider overdose deaths from any other substance such as antidepressants, nor do we consider poisoning by alcohol. The CDC WONDER database suppresses entries with fewer than 10 deaths.

Crude rates for a specific demographic category are defined as the number of fatalities of that category divided by the mid-year population of the same category, multiplied by the typical factor of 100,000. Since WONDER lists male and female genders only, we use a binary gender classification. We further stratify our data by the four Census Regions listed in WONDER: the Northeast (Region 1), the Midwest (Region 2), the South (Region 3) and the West (Region 4). Since data are also catalogued by state plus the District of Columbia, we finally examine all 51 jurisdictions and refer to them collectively as “states”. The WONDER database also lists four racial categories (White, Black or African American, Asian or Pacific Islander, American Indian or Alaska Native) but we focus our analysis on White and Black or African American racial categories since only these two were associated with statistically significant numbers of fatalities. The CDC specifies that racial information is derived from the death certificate of the decedent as reported by the funeral director and as provided by the next of kin or on the basis of observation [12]. Multiple race decedents are assigned to the race that most closely aligns with the single races listed above for a more compact classification.

For each geographical or racial category we consider the crude rates for females and males and derive the male-to-female crude ratio by dividing the two. We identify 2013–2019 trends and determine whether 2020 data are statistical outliers. To do this, we first fit the 2013–2019 data to a quadratic form, which allows for possible non-monotonic behaviors. We then calculate the standard deviation of the residuals between the fitted trends and the data across 2013–2019. If the residual corresponding to 2020 is three times beyond the value of the standard deviation, the 2020 value is considered an outlier. All of our data analyses were done using pandas, an open source Python package that is part of the Anaconda distribution. Maps have been created with the Python package geopandas.

## Results

### 1. Nationwide

**Fig 1:**
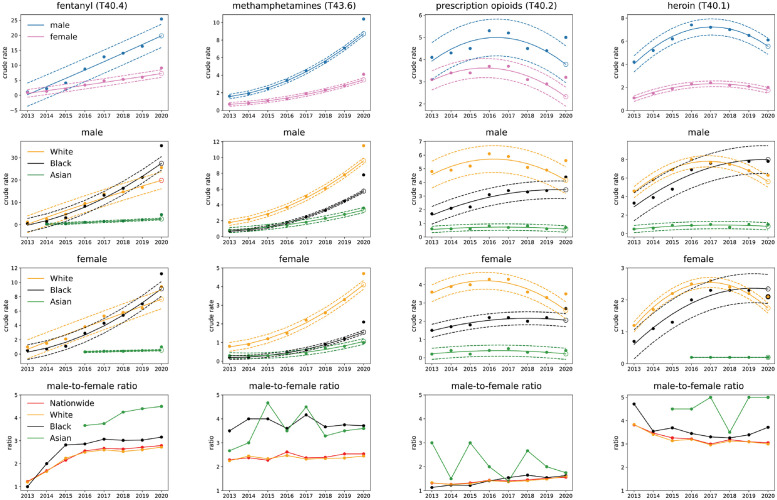
Nationwide gender and race stratified crude rate trends for synthetic opioids (fentanyl, T40.4), psychostimulants with abuse potential (methamphetamines, T43.6), semi-synthetic opioids (prescription opioids, T40) and heroin (T40.1). Solid lines in the upper three rows represent the 2013–2019 quadratic regression; dotted lines correspond to values above and below the regression by three times the standard deviation calculated from 2013–2019 data; the open circle is the 2020 projection. Top row: results are stratified by gender. Note the increasing 2013–2019 trend and the 2020 value exceeding projections for fentanyl (T40.4) and methamphetamines (T43.6) for both genders; crude rates for prescription opioids (T40.2) and heroin (T40.1) have been decreasing but rose again in 2020, with significant rises especially in prescription opioids (T40.2) for both genders. Second (third) row from top: results are stratified by race for males (females). For fentanyl and heroin the Black male crude rate surpassed that of Whites in 2018. Last row: Male-to-female ratio for all races. The ratio for Blacks is higher than the ratio for Whites in all categories except for prescription opioids (T40.2). We include results for Asians in years when data is available; although crude rates are the lowest in the nation, this is the race with the largest male-to-female ratio in all drug categories.

Nationwide, fentanyl (T40.4) and methamphetamines (T43.6) fatal overdose crude rates increase monotonically for both genders over the 2013–2019 time frame as shown in Fig 1. For both drug classes and genders, the 2020 values are statistical outliers, as they are in excess of three standard deviations of the residuals above the quadratic regression fit. In particular, fentanyl (T40.4) male fatal overdose rates in 2020 exceed projections by 30%; female rates by 28%. Methamphetamine (T43.6) male fatal overdose rates exceed projections by 19%; female rates by 18%. The fentanyl (T40.4) male-to-female fatal overdose ratio increases in an approximate linear manner from 2013 to 2016, settling around 2.6 and slightly increasing thereafter. At 2.8, the 2020 value is consistent with this trend. For methamphetamines (T43.6) the male-to-female ratio is relatively uniform over the 2013–2020 period and fluctuates between 2.3 and 2.6. This suggests that while 2020 saw a dramatic nationwide increase in the number of deaths due to fentanyl and methamphetamines, the impact was similar across genders.

Stratifying the data by race reveals racial disparities for fentanyl fatal overdose rates (T40.4) that can be seen in Fig 1. Crude rates for Black and White males were comparable until 2018 (even slightly higher for White males); the Black male fatal overdose rate exceeded that of White males for the first time in 2018. In 2020 fentanyl (T40.4) nationwide crude rates for Black males (35.4 fatalities per 100,000) exceeded that of White males (25.6 fatalities per 100,000) by 38%. Similar trends are observed for females: the crude rate for White females first surpassed that of Black females in 2019, and in 2020 the crude rate for Black females (11.2 fatalities per 100,000) was 19% larger than that of White females (9.4 fatalities per 100,000). Racial disparities are also observed among the male-to-female ratio of crude rates. All ratios grow until 2015 after which they stabilize; however, the Black male-to-female ratio increased more than the 2.6 White male-to-female ratio reaching 3.2 in 2020.

Fatal overdose rates due to methamphetamines (T43.6) are higher among White individuals than Black individuals throughout the entire 2013–2020 period. However the male-to-female ratio for Black persons is always much higher than that for White persons. In 2020 for example, the male-to-female ratio for Black persons is 3.8, for White persons it is 2.4.

Nationwide trends for prescription opioids (T40.2) and heroin (T40.1) overdose death rates display non-monotonic trends: mortality increased between 2013 and 2016 for both genders, and began decreasing in 2017. However, while the 2020 rates for heroin (T40.1) remain consistent with downward predictions, those for prescription opioids (T40.2) show a statistically significant upward shift, with males surpassing them by 32% and females by 37%. The male-to-female fatal overdose ratio is relatively stable at around 3.1 for heroin (T40.1), and is slowly increasing for prescription opioids (T40.2) reaching 1.6 in 2020, the lowest of all drug categories.

Stratification by race shows that nationwide Black mortality rates did not decline for either prescription opioids (T40.2) or heroin (T40.1) over the 2013–2019 interval but instead increased for both genders. In particular, for heroin (T40.1) the nationwide Black male fatal overdose rate was less than that of White males until 2017. After the two reached similar levels in 2018, the crude rate among Black males kept increasing at a higher rate than among White males, until in 2020 the crude rate for Black males exceeded that of White males by 24%. For prescription opioids (T40.2) instead, nationwide fatal overdose rates remained larger for White persons than for Black persons throughout the entire 2013–2020 period. The male-to-female ratio is comparable for the two races, for both heroin (T40.1) and prescription opioids (T40.2).

Taken together, these results suggest that the most significant changes nationwide are the statistically significant 2020 increases in mortality due to fentanyl and methamphetamines and the resurgence in prescription opioid deaths for both genders. Despite a non-statistically significant increase in 2020, heroin fatal overdose rates have been declining nationwide since 2017, but not among Black individuals. Nationwide fatal overdose rates for Black persons have surpassed those of White persons for fentanyl for both genders, and for heroin for males.

### 2. Census regions

Our Census region analysis shows that overdose deaths have been steadily increasing since 2013 for fentanyl (T40.4) and for methamphetamines (T43.6) for both genders in all regions (Northeast, Midwest, South, West). Overall, fentanyl (T40.4) crude rates were highest in the Northeast for both genders whereas the highest methamphetamine (T43.6) crude rates for both genders are observed in the West. Peak fatal overdose rates have been reached for prescription opioids (T40.2) and for heroin (T40.1) in all regions with the exception of the West where heroin-induced deaths have been steadily increasing since 2013.

Stratification by race leads to a more differentiated picture. During the early period of our 2013–2020 analysis, crude rates due to fentanyl (T40.4) were higher among White males than among Black males in all four Census regions. However by 2020 a “switch” had occurred and crude rates among Black males had surpassed those of White males in all four regions. For fentanyl (T40.4) the switch occurred for the first time in the Midwest in 2016, with the Black-White gap widening in subsequent years. In 2020 crude rates are 60 fatalities per 100,000 Black males and 25 per 100,000 White males. The overall crude rate (without stratifying by race) in the Midwest is 29 fatalities per 100,000 males. Black males in the Midwest are also the demographic category that is associated with the largest fatal fentanyl (T40.4) crude rate in the United States in 2020. In other regions the Black-White male switch is more recent and occurred around 2019 or 2020.

A similar trend is observed for fentanyl-induced overdoses among females. In 2020, the crude rate in the Midwest is 20 deaths per 100,000 Black females whereas the same rate is 10 among White females. The overall crude rate in the Midwest for female deaths is 11 fatalities per 100,000. The vast difference in these numbers (60 for Black males vs 25 for White males; 20 for Black females and 10 for White females) reveals large racial disparities that are not readily apparent if only overall crude rates are considered.

Crude rates for methamphetamines are also increasing in all Census regions for both races and genders. However, mortality among White males and females remains larger than among Black males and females, respectively, over the entire 2013–2020 timeframe except for the West. Here, overdose crude rates among Black males surpassed those of White males in 2016; for females the switch occurred in 2018. The racial gap in the West has increased in subsequent years, and in 2020 it was 25 fatalities per 100,000 Black males and 17 per 100,000 White males; among Black females the rate was 8 per 100,000 and among White females it was 6 per 100,000.

The Northeast and the Midwest exhibited the highest heroin fatal overdose rates; in both regions male Black mortality has increased or remained stable since 2013, similarly for Black females in the Northeast. Conversely, heroin fatal overdose rates for Whites in these regions declined, especially in the Midwest. Finally, in the Northeast prescription opioid crude rates are decreasing for White males and females, but are increasing for their Black counterparts. Relevant plots for all four Census regions (Northeast, Midwest, South and West) are shown in Fig A-D in S1 Text.

### 3. States

Concurrent stratification by gender, race, drug type and state does not allow for a consistent analysis over the 2013–2020 range due to insufficient data for many jurisdictions and drug classes, especially in the earlier years. Thus, in this section, we pool yearly overdose deaths for all drug types (fentanyl T40.4, methamphetamines T43.6, prescription opioids T40.2, heroin T40.1), regardless of gender and race, in each state and analyze the corresponding trends. For almost all states there is sufficient data to analyze fentanyl-induced mortality (without considering race and gender) over the entire 2013–2020 period. A similar state by state analysis is not possible for the other drug classes, since the mortality is comparatively lower, especially in the earlier years. We could instead compare 2019 to 2020 crude rate data for each state, drug type and gender (without considering race), and 2019 to 2020 crude rate data for both genders in each state and for each drug type, given there is sufficient data in the latter two years of our 2013–2020 dataset for a significant statistical analysis.

Predictions for 2020 as extrapolated from 2013–2019 trends are obtained using the same procedure described in the Methods section and used for the Nationwide and Census regions. Upon combining all drug-induced deaths in each state, we find that 2020 fatal overdose rates exceed predictions for all states except for Connecticut, the District of Columbia, Hawaii, Idaho, Iowa, Maryland, Michigan, Montana, Nebraska, Pennsylvania and South Dakota. There was not enough data for North Dakota. Conversely, for New Jersey and Delaware overdose crude rates were less than what predicted on the basis of 2013–2019 data.

Data is sufficiently abundant in most states to also analyze fentanyl (T40.4)-induced deaths for all races and genders; the only exceptions are Alaska, Hawaii, Montana, Nebraska, North Dakota, South Dakota, Wyoming. We find that the 2020 data exceeds predictions in a statistically significant manner for all remaining 43 states and the District of Columbia except for Connecticut, Maine, Maryland, Massachusetts, Michigan, Montana, Ohio, Nebraska, Pennsylvania and Vermont. Similarly to what reported above for all drugs pooled together, crude rates were less than predictions based on 2013–2019 data in New Jersey and Delaware.

**Fig 2:**
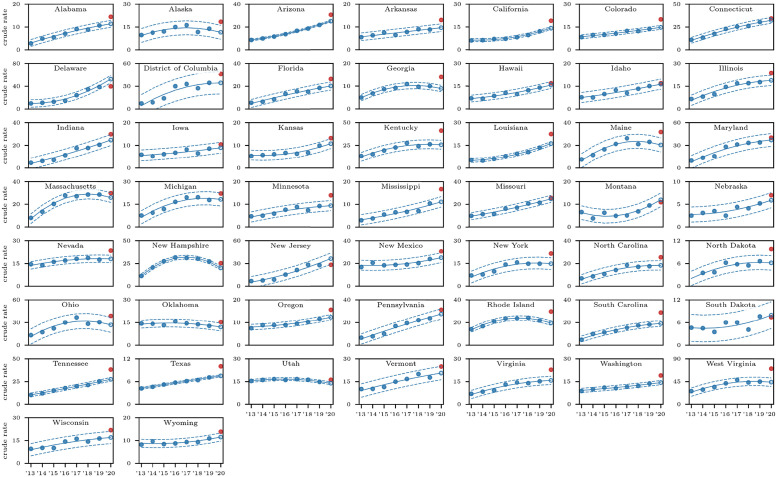
State-by-state fatal overdose trends for all races, all genders and all drug types, i.e. fentanyl (T40.4), methamphetamines (T43.6), prescription opioids (T43.6) and heroin (T40.1). Solid lines represent the 2013–2019 quadratic regression; dotted lines correspond to values three times above and below the regression by three times the standard deviation calculated from 2013–2019 data; the open circle is the 2020 projection, the filled red dot is the actual data. Note that in most states (but not all) an increasing 2013–2019 trend and that the 2020 value exceeds projections. In New Jersey and Delaware, crude rates were less than those predicted based on the 2013–2019 quadratic regression. There is insufficient data for North Dakota.

**Fig 3:**
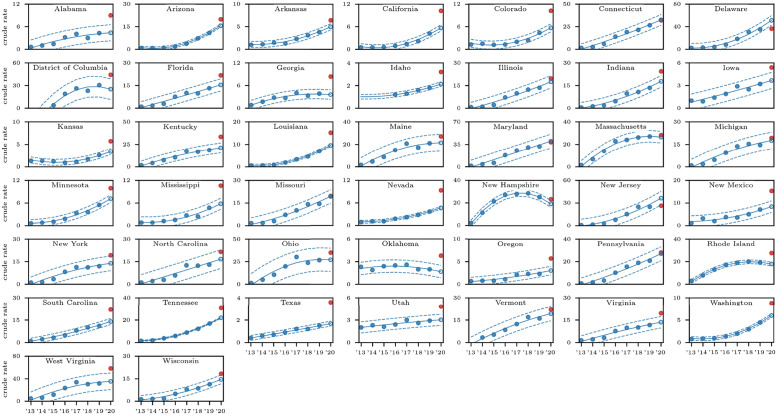
State-by-state fatal overdose trends induced by fentanyl (T40.4) for all races and all genders. Solid lines represent the 2013–2019 quadratic regression; dotted lines correspond to values three times above and below the regression by three times the standard deviation calculated from 2013–2019 data; the open circle is the 2020 projection, the filled red dot is the actual data. In most (but not all) states trends mirror those observed for overall fatal overdoses shown in Fig 2. There is insufficient data for Alaska, Hawaii, Montana, Nebraska, North Dakota, South Dakota, Wyoming.

Crude rates for deaths due to all drug types and for fentanyl-induced deaths are shown in Figs 2 and 3 respectively. Most notably, the overall 2013–2019 fentanyl (T40.4) trend mirrors that of the pooled drugs in most states, with crude rates increasing in both cases. Exceptions are Oklahoma and Utah where pooled drug overdose crude rates are decreasing but fentanyl (T40.4) crude rates are increasing; New Hampshire and Rhode Island where both rates are decreasing and the District of Columbia, Georgia, Maine, Massachusetts and Ohio where both rates reach a plateau.

States with the largest gap between actual 2020 crude rates due to all drug types and predictions based on 2013–2019 data are Alaska, Kentucky, West Virginia (all at +61%), whereas for fentanyl-induced deaths, the largest 2020 gap between actual crude rates and predictions are recorded in Oklahoma (+125%), Texas (+115%), Alabama (+106%).

**Fig 4:**
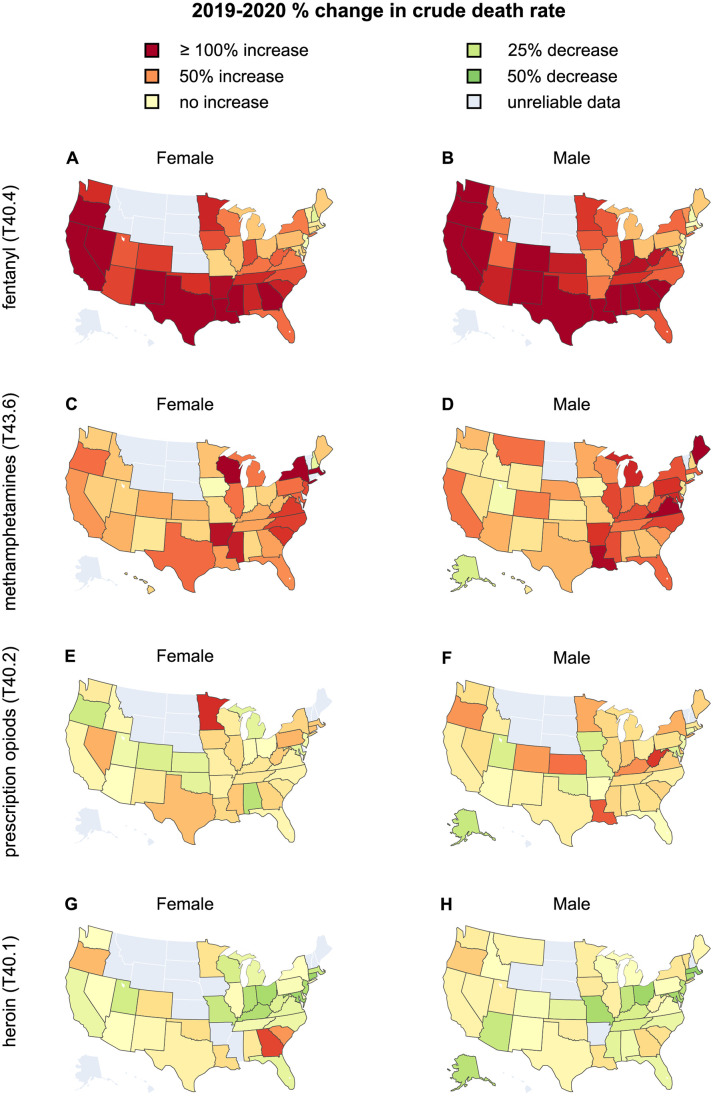
Percentages of change to the crude rate between 2019 and 2020 stratified by gender for fentanyl (T40.4, panels A, B), methamphetamines (T43.6, panels C, D), prescription opioids (T40.2, panels E, F), and heroin (T40.1, panels G, H), in the United States. In states shown in dark red, crude rates more than doubled while green indicates that crude rates decreased between 2019 and 2020.

In Fig 4 we show geographical variations in the male and female crude rates in 2019 and 2020 within the United States. Large increases for fentanyl (T40.4) and methamphetamines (T43.6) crude rates are observed in almost all states, consistent with national and Census region trends. The picture is more varied for heroin (T40.1) and prescription opioids (T40.2) for which a decrease in overdose deaths is observed across several states. Some display large gender disparities, such as Georgia, where heroin crude rates for females increased by more than 100% but only slightly for males between 2019 and 2020. Similar gender patterns are observed for prescription opioid (T40.2) deaths in Minnesota. Prescription opioid (T40.2) crude rate increases from 2019 to 2020 are instead much larger for males than females in West Virginia and Louisiana.

**Fig 5:**
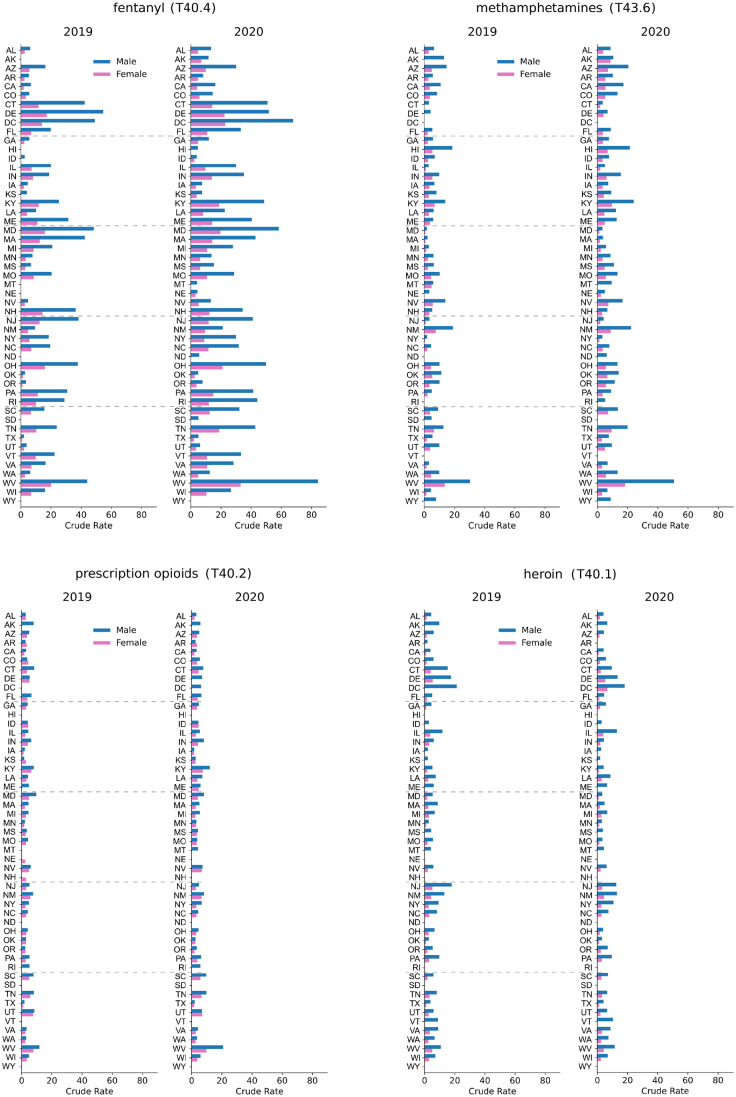
Gender-stratified, crude rates across the United States associated with fentanyl (T40.4), methamphetamines (T43.6), prescription opioids (T40.2), heroin (T40.1) for the years 2019 and 2020. Note the gender imbalances and increase in mortality for West Virginia from 2019 to 2020 for all drug categories except heroin.

Gender-stratified crude rates for 2020 and 2019 are shown for all states in Fig 5. For all drug classes examined except heroin (T40.1), the largest 2020 crude rates for males and females are in West Virginia, followed by Kentucky for methamphetamines (T43.6) and prescription opioids (T40.2); for fentanyl (T40.4) the second largest crude rates for males and females are recorded in the District of Columbia. For heroin, the largest male and female crude rates are in the District of Columbia followed by Delaware.

Upon pooling all drugs and maintaining a gender stratification, we also find that fatal overdose rates increased everywhere in 2020 compared to 2019 except for New Hampshire and South Dakota for both genders. For males, drug overdose deaths also decreased in Delaware, for females in Montana and New Jersey. The combined male-to-female overdose ratio increased in 36 states in 2020 compared to 2019. Significant upward shifts occurred in Kentucky, West Virginia and Colorado where deaths by overdose rate rose more for males than for females. There were also 13 states where the male-to-female ratio decreased or remained comparable to 2019. The most significant downward shifts in the male-to-female ratios were recorded in Alaska (+16% males, +82% females) and Delaware (-3% males, +20% females), indicating relatively higher drug overdose deaths among females in these states.

**Fig 6:**
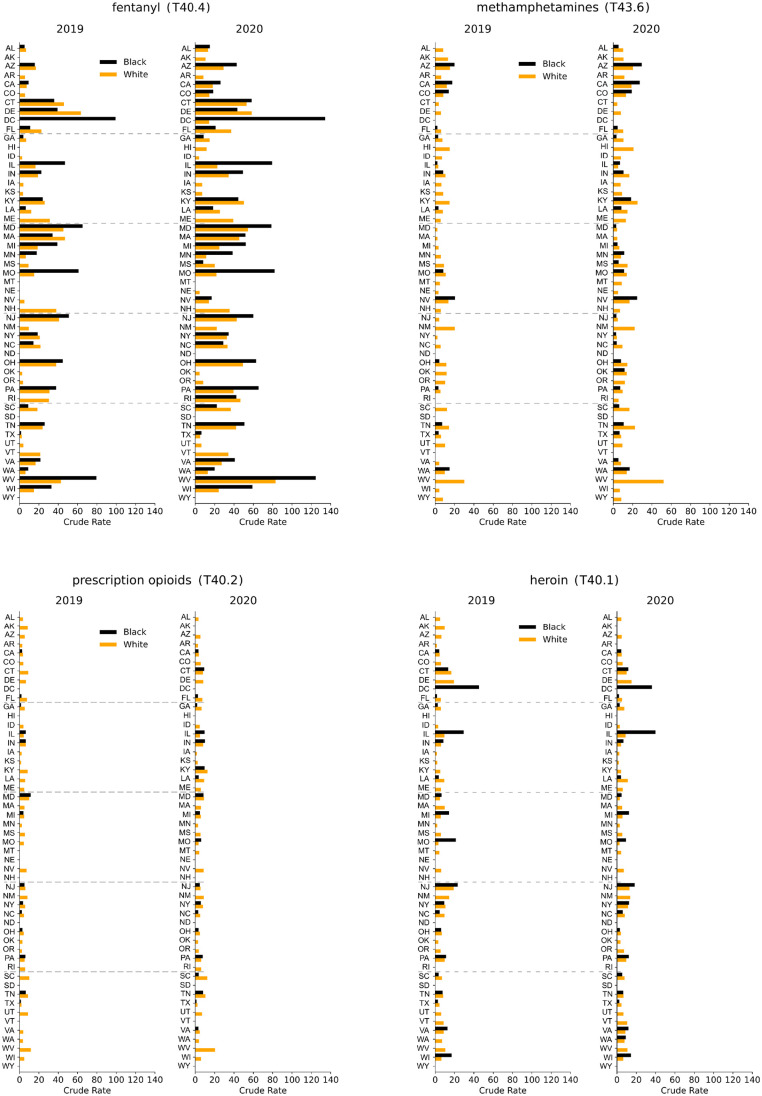
Crude rates for Black and White males across the United States associated with fentanyl (T40.4), methamphetamines (T43.6), prescription opioids (T40.2), heroin (T40.1) for the years 2019 and 2020. Note the racial imbalances and the increase in mortality in the District of Columbia for fentanyl and heroin.

As observed above, if only gendered overdose deaths are considered, the largest 2020 fentanyl (T40.4) crude rates are in West Virginia (84 fatalities per 100,000 males and 33 fatalities per 100,000 females). Upon differentiating by race, dramatic disparities emerge, primarily in the District of Columbia, as can be seen in Fig 6. Here, until 2015 there was an insufficient number of fentanyl (T40.4) overdose deaths for either gender or race, however in 2016 a large spike of Black male deaths emerged (57 fatalities per 100,000). Mortality kept increasing over the years until in 2020 a record crude rate of 134 fatalities per 100,000 males (+35% with respect to 2019) and 43 fatalities per 100,000 females (+56% with respect to 2019) were recorded. Both are the highest in the nation in 2020 and 2019. The only significant fentanyl (T40.4) datapoint in the District of Columbia for White persons over the entire 2013–2020 period is a crude rate of 14 fatalities per 100,000 males in 2020. This implies that the vast majority of fentanyl (T40.4) victims in the District of Columbia are Black males, and that in 2020 there were 9.4 times more fatalities among Blacks than among Whites. Ever since fentanyl (T40.4) deaths were first tallied in the District of Columbia in 2016, Black male crude rates have been larger than those of White (and Black) males in West Virginia, the state with the largest overall male fentanyl mortality.

Large racial disparities are also observed for male heroin deaths in the District of Columbia. Although Black male mortality in the District of Columbia peaked at 55 fatalities per 100,000 in 2016, the 2020 male crude rate is still among the highest in the nation at 36 fatalities per 100,000. There is insufficient data for White males. This pattern of elevated Black mortality and not statistically significant White overdose deaths is consistent over the entire 2013–2020 period. The state with the largest Black male crude rate in 2020 is Illinois, at 40 deaths per 100,000, whereas the corresponding rate for White males is 9 deaths per 100,000, representing a 4.4 Black to White male ratio. Throughout 2013–2020 heroin crude rates in Illinois have been higher for Blacks than for Whites; while rates kept increasing or stabilized for Blacks, they began declining for Whites in 2017.

Other states with large racial gaps for fentanyl overdose deaths during 2020 are in the Midwest, and include Illinois, Minnesota, and Missouri, for both genders, consistent with 2019 data. The male racial gap increased in 2020 compared to 2019 in all states for which data is available except for West Virginia, Missouri and Kentucky, where it decreased slightly; for women the racial gap increased in most states, but decreased in ten mostly southern states including South Carolina, Louisiana and Florida. Conversely, in 2020 White males display larger crude rates than Black males for methamphetamines (T43.6) and prescription opioids (T40.2) in several southern states, including Georgia, South Carolina and Florida, consistent with 2019 data.

## Discussion

The year 2020 saw increases in drug-induced mortality across the United States compared to previous years. Mortalities for fentanyl and methamphetamines exceed projections from 2013–2019 (which already anticipated large increases) for both genders. Overall, 2013–2020 saw an explosion in overdose deaths nationwide with fatal overdose rates by synthetic opioids increasing by 2,209% for males and by 991% for females. Methamphetamine fatal rates follow similar patterns (+550% for males, +486% for females). Beginning in 2017, natural and semi-synthetic opioid overdose rates began decreasing for both genders, followed by a large, statistically significant resurgence in 2020; a similar pattern is observed for heroin for which the limited 2020 increase was not statistically significant. Male mortality exceeds female mortality in all states; the 2020 male-to-female overdose ratio nationwide was highest for heroin (3.1), followed by synthetic opioids (2.8), methamphetamines (2.6) and natural and semi-synthetic opioids (1.6). This is consistent with data from previous years and with the hypothesis that females preferentially consume (and hence die from) natural and semi-synthetic opioids contained in prescription pain-killers which are easier and safer to acquire than street drugs [7].

A more nuanced picture emerges after racial stratifications are included. For example, crude rates for fentanyl overdose deaths among Black males and females have increased more than among Whites over the past few years; in 2018, and for the first time, Black male crude rates reached and later surpassed those of White males. Between 2013 and 2020 fentanyl crude rates for Black males rose nationwide by 6,980%; those for Black females by 2,140%. These increases are much larger than those recorded for the population at large (+2,209% for males; +991% for females) and for Whites (+ 2,033% for males; +840% for females). Similarly, the crude rate of heroin overdose deaths for the male and female population at large is decreasing within the United States and among White persons, however it is still increasing among Black males. These findings mirror other studies that have focused on nationwide racial disparities in drug overdose deaths [13–15].

Combined gender, race and geographical stratifications are important as they may shift our perception of the most at risk groups [16, 17]. For example, in 2020 the highest crude rate for fentanyl deaths is in West Virginia at 84 deaths per 100,000 persons. In the District of Columbia it is 68 deaths per 100,000 persons. However, as discussed above, a deeper analysis reveals that the fentanyl epidemic is much more severe for Black males in the District of Columbia with 134 cases per 100,000 Black males; the White male crude rate is 14 deaths per 100,000, indicating significant disparity between the two races. The fentanyl crude rate for Black females in the District of Columbia is 43 deaths per 100,000; there is insufficient data for White females. This imbalance is also evident in West Virginia where the fentanyl crude rate is 125 deaths per 100,000 Black males and 83 deaths per 100,000 White males. The overall crude rate in West Virginia, at 84 deaths per 100,000, is comparable to that of White males because of the smaller percentage of Black individuals residing in the state, which heavily weighs the crude rate towards that of White individuals. Our results reinforce the importance of including racial stratifications as they provide a better gauge of the severity of the drug epidemic.

Fentanyl crude rates for Black males exceeded those of White males in many other states where there is sufficient 2020 data to allow for racial stratification. Large disparities are seen in Illinois, Minnesota and Missouri. The trend is reversed in most Southern states where male crude rates remain higher for Whites than Blacks. The highest 2020 crude rate for Black females is in the District of Columbia, at 43. Deaths among White females are too low to yield a significant statistic. Large racial disparities also emerge for male heroin overdose deaths in Illinois, Missouri and Michigan. Another important finding for 2020 is that male overdose crude rates for all drug types and genders increasing more than female crude rates almost everywhere except for Alaska, where crude rates rose by 16% for males but by a very large 82% for females. In Delaware male crude rates decreased by 3% but female mortality rose by 20%.

Why do some jurisdictions display such wide racial and gender disparities, while in others overdose rates are more uniform? One step further in our analysis, and an attempt to resolve this question, would be to quantify other factors such as poverty, inequality indices, income, unemployment, drug availability, access to prevention and treatment in various regions, and determine which of these correlate the most with the observed crude rates. Such an analysis could help provide a clearer picture of the drug epidemic and help prioritize interventions. Whatever the cause, the explosion in drug-induced deaths during the 2013–2020 time frame underlines the need for allocating more resources and expanding programs that can help prevent and treat addiction and that can reduce drug overdose deaths [18]. These measures should be tailored to specific sociodemographic groups, by race, gender, geography or age in order to be most effective, especially given the non-uniform gender and racial trends outlined in this and other studies [13–15].

Particularly worrisome is the increase in fentanyl deaths, which is 50 to 100 times stronger than morphine and which carries a high risk for addiction. One of the most urgent and life-saving measures would be to educate the general population, especially the young, on the dangers and potency of fentanyl, raising awareness that doses as small as a few milligrams might lead to death. First and long-time users should also be made aware of the possibility of less potent drugs being tainted with fentanyl in unknown, sometimes fatal, doses. Especially in recent years, powdered form fentanyl is being mixed with prescription benzodiazepines or amphetamines, such as Xanax or Adderall, or with heroin, cocaine and methamphetamines. This allows drug dealers to increase profit margins and the euphoric effects of their products. At times, users keen on experimentation engage in the practice as well. Other useful measures to curb the drug overdose epidemic would be to expand availability and access to naloxone, an opioid antagonist that can rapidly reverse the effects of opioids. Widespread training on the proper administration of naloxone could also reduce overdose deaths. Promoting the use of fentanyl test strips among drug users may allow them to detect the presence of fentanyl in drug samples, also reducing the risk of fentanyl overdoses.

### Limitations

Our analysis is based on four drug categories selected from the CDC WONDER database: fentanyl, prescription opioids, heroin, methamphetamines (respectively T40.4, T40,2, T40.1, T43.6). They are the ones with the largest crude rates, allowing us to follow temporal trends and stratify deaths as much as possible by race and gender. Other categories such as T40.3 (methadone) or T40.5 (cocaine) were not included in this study due to lower mortality rates; deaths from these categories may display different dynamics. We did not consider Hispanic origin, nor did we consider other races such as Asian or Pacific Islander (except in Fig 1), or American Indian or Alaska natives. While still much lower than the mortality rates of Black and White individuals, both Asian and American Indian mortality rates have been increasing in recent years. We did not include any stratification by age. Since WONDER suppresses entries where the number of deaths is less than 10, data is not available for some combinations of states, genders and races with extremely low fatalities (typically females). Finally, if multiple drugs are found in a person who overdosed, the death is tallied in all respective categories, resulting in multiple counts.

### Public health implications

There are several public health implications stemming from our work. First, although identifying the underlying causes of increasing overdose deaths in 2020 is beyond the scope of this work, it is reasonable to assume that pandemic-related anxieties [19–22], the greater availability of cheaper (and potentially contaminated) drugs, and the ease of ordering drugs online [23] have contributed to rising mortality. Isolation may have also made it easier for users to abuse drugs alone, leading to fewer interventions by partners and reduced access to emergency resources in case of fatal or non-fatal overdose. Since decreasing prices, drug contamination, and easier off-the-street procurement methods may persist in a post-pandemic world, overdose rates may remain high in the coming years. Thus, efforts devoted to curtailing online and mail-order drug transactions should be expanded. Second, we find that stratifying by race and gender may lead to surprising differences between demographic groups. This to true even for overdose deaths occurring prior to the pandemic. Thus, in order to more efficiently implement prevention and harm reduction initiatives one must first understand the internal dynamics and barriers facing diverse groups vis-à-vis drug addiction [24–26], so that these initiatives can be optimally adapted to specific group needs. Given that Black males are often the most-at-risk category, and that their fatal overdose rates are increasing dramatically, dedicated programs should be tailored to this specific demographic [27] through educational preventive initiatives, awareness of fatal overdose risks associated to fentanyl, naloxone distribution, access to treatment programs and post-detox support. Although we did not include an age-stratified analysis, age-specific interventions and awareness programs should also be considered [28].

Finally, in some states the 2020 fatal overdose crude rates did not significantly exceed those recorded in 2019. In Delaware and New Jersey crude rates for all drugs combined and for fentanyl (T40.4) actually decreased. In other states, primarily in the Northeast, the 2013–2019 trends reveal non-monotonic behavior. It would be interesting to learn the main drivers of these trends and to distill which measure, if any, led to peak or flattened crude rates and whether they impacted all races.

Data and codes for statistical analysis, curve fitting and for deriving the 3σ bandwidths are available at this link: https://github.com/dorsogna/drugs

## Supporting information

S1 Text Previous year trends as well as 2020 predictions and actual overdose death rates for the four Census regions of the United States. (PDF)

## References

[1] Ahmad FB, Cisewski JA, Rossen LM, Sutton P. Provisional drug overdose death counts. National Center for Health Statistics. 2023. https://www.cdc.gov/nchs/nvss/vsrr/ drug-overdose-data.htm

[2] Friedman J, Beletsky L, Schriger DL. Overdose-related cardiac arrests observed by emergency medical services during the US COVID-19 epidemic. JAMA Psychiatry. 2021;78: 562–564. doi: 10.1001/jamapsychiatry.2020.4218 33270086 PMC7716252

[3] United Nation Office on Drugs and Crime (UNODC). COVID-19 and the drug supply chain: from production and trafficking to use, Vienna 2020. https://www.unodc.org/documents/data-and-analysis/covid/Covid-19-and-drug-supply-chain-Mai2020.pdf

[4] Hedegaard H, Miniño, AM, Spencer, MR. Warner, M. Drug overdose deaths in the United States, 1999–2020. National Center for Health Statistics. Data Brief, no. 428. https://www.cdc.gov/nchs/products/databriefs/db428.htm

[5] Spencer MR, Miniño AM, Warner M. Drug overdose deaths in the United States, 2001–2021. National Center for Health Statistics. Data Brief, no 457. https://www.cdc.gov/nchs/products/databriefs/db457.htm

[6] Mattson CL, Tanz LJ, Quinn K, Kariisa M, Patel P, Davis NL. Trends and geographic patterns in drug and synthetic opioid overdose deaths—United States, 2013–2019. MMWR Morb Mortal Wkly Rep. 2021;70: 202–207. doi: 10.15585/mmwr.mm7006a4 33571180 PMC7877587

[7] Ho JY. Cycles of gender convergence and divergence in drug overdose mortality. Popul Dev Rev. 2020;46: 443–470. doi: 10.1111/padr.12336 33583972 PMC7880043

[8] Becker JB, Mc Clellan ML, Reed BG. Sex differences, gender and addiction. J Neurosci Res. 2017;95: 136–147. doi: 10.1002/jnr.23963 27870394 PMC5120656

[9] Larochelle MR, Slavova S, Root ED, Feaster DJ, Ward PJ, Selk SC, et al. Disparities in opioid overdose death trends by race/ethnicity 2018–2019 from the HEALing Communities Study. Am J Public Health. 2021;111: 1851–1854. doi: 10.2105/AJPH.2021.306431 34499540 PMC8561170

[10] O’Donnell J, Tanz LJ, Gladden M, Davis NL, Bitting J. Trends in and characteristics of drug overdose deaths involving illicitly manufactured fentanyls–United States, 2019–2020. MMWR Morb Mortal Wkly Rep. 2021;70: 1740–1746. doi: 10.15585/mmwr.mm7050e3 34914673 PMC8675656

[11] Friedman J, Hansen H. Evaluation of increases in drug overdose mortality rates in the US by race and ethnicity before and during the COVID-19 pandemic. JAMA Psychiatry. 2022;79: 379–381. doi: 10.1001/jamapsychiatry.2022.0004 35234815 PMC8892360

[12] Wide-ranging online data for epidemiologic research (WONDER). CDC, National Center for Health Statistics 2020. http://wonder.cdc.gov

[13] Furr-Holden D, Milam AJ, Wand L, Sadler R. African Americans now outpace Whites in opioid-involved overdose deaths: A comparison of temporal trends from 1999 to 2018. Addiction. 2021;116: 677–683. doi: 10.1111/add.15233 32852864

[14] Han B, Cotto J, Etz K, Einstein EB, Compton WM, Volkow ND. Methamphetamine overdose deaths in the US by sex and race and ethnicity. JAMA Psychiatry. 2021;78: 564–567. doi: 10.1001/jamapsychiatry.2020.4321 33471025 PMC8100861

[15] Han B, Einstein EB, Jones CM, Cotto J, Compton WM, Volkow ND. Racial and ethnic disparities in drug overdoses in the US during the COVID-19 pandemic. JAMA Netw Open. 2022;5: e2232314. 36125815 10.1001/jamanetworkopen.2022.32314 PMC9490498

[16] Friedman J, Hansen H. Far From a “White Problem”: Responding to the overdose crisis as a racial justice issue. Am J Public Health. 2022;112: S30–S32. doi: 10.2105/AJPH.2021.306698 35143272 PMC8842209

[17] Hulsey JN. Toward improved addiction treatment quality and access for Black patients. Am J Public Health. 2022;112: S21–S23. doi: 10.2105/AJPH.2021.306664 35143277 PMC8842210

[18] Humphreys K, Shover CL, Andrews CM, et al. Responding to the opioid crisis in North America and beyond: Recommendations of the Stanford-Lancet Commission. Lancet. 2021;399: 555–604. 10.1016/S0140-6736(21)02252-2 PMC9261968 35122753

[19] Cartus AR, Li Y, Macmadu A, Goedel WC, Allen B, Cerda M, et al. Forecasted and observed drug overdose deaths in the US during the COVID-19 pandemic in 2020 JAMA Netw Open. 2022;5: e223418. doi: 10.1001/jamanetworkopen.2022.3418 35311967 PMC8938716

[20] Sher L. The impact of the COVID-19 pandemic on suicide rates. QJM. 2020;11: 707–712. doi: 10.1093/qjmed/hcaa202 32539153 PMC7313777

[21] Woolf SH, Chapman DA, Sabo RT, Weinberger DM, Hill L. Excess deaths from COVID-19 and other causes. JAMA. 2020;324: 510–513. 32609307 10.1001/jama.2020.11787 PMC7330820

[22] Substance Abuse and Mental Health Services Administration (SAMSHA). Key substance use and mental health indicators in the United States: Results from the 2021 National Survey on Drug Use and Health. HHS publication no. PEP22-07-01-005, NSDUH Series H-57. 2022. https://www.samhsa.gov/data

[23] Shelley L. Fentanyl, COVID-19, and Public Health. World Med Health Policy. 2020;12: 390–397.

[24] Brady KT, Randall CL. Gender differences in substance use disorders, Psychiatr Clin North Am. 1999;22: 241–252. 10385931 10.1016/s0193-953x(05)70074-5

[25] Hansen H, Jordan A, Plough A, Alegria M, Cunningham C, Ostrovsky A. Lessons for the opioid crisis—Integrating social determinants of health into clinical care. Am J Public Health. 2022;112: S109–S111. doi: 10.2105/AJPH.2021.306651 35349328 PMC8965192

[26] Lopez AM, Thomann M, Dhatt Z, Ferrera J, Al-Nassir M, Ambrose M, et al. Understanding racial inequities in the implementation of harm reduction initiatives. Am J Public Health. 2022;112: S173–S181. doi: 10.2105/AJPH.2022.306767 35349311 PMC8965181

[27] Blackwood CA, Cadet JL. COVID-19 Pandemic and fentanyl use disorder in African Americans. Front Neurosci. 2021;15: 707386. doi: 10.3389/fnins.2021.707386 34489626 PMC8417443

[28] Friedman J, Godvin M, Shover CL, Gone JP, Hansen H, Schriger DL. Trends in drug overdose deaths among US adolescents, January 2010 to June 2021. JAMA. 2022;327: 1398–1400. doi: 10.1001/jama.2022.2847 35412573 PMC9006103

