# Supplementary material for: Fentanyl-driven acceleration of racial, gender and geographical disparities in drug overdose deaths in the United States
Source: PLOS Glob Public Health. 2023 Mar 22;3(3):e0000769. doi: 10.1371/journal.pgph.0000769 (PMC10032521; doi:10.1371/journal.pgph.0000769)
Supplement: S1 Text — (PDF) [file pgph.0000769.s001.pdf]

S1 Text

Below we show the 2013-2019 trends as well as 2020 predictions and actual overdose death rates for the four Census regions of the United States. Methods are similar to those discussed in the Nationwide results section.

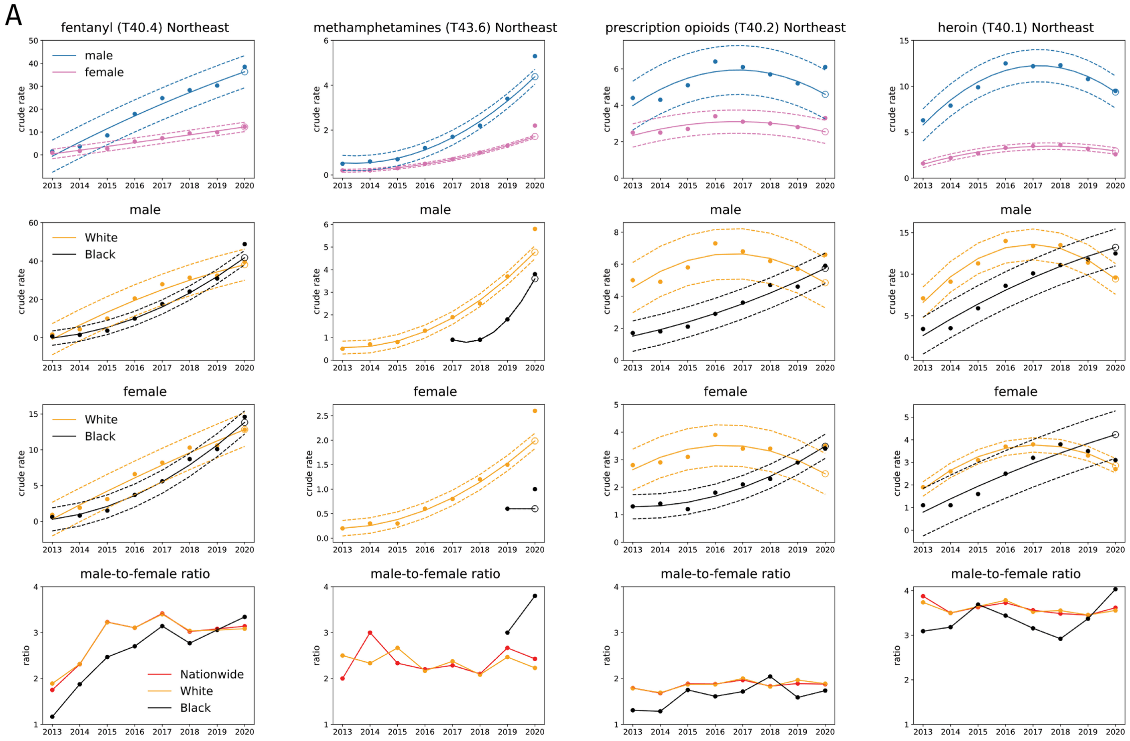

B

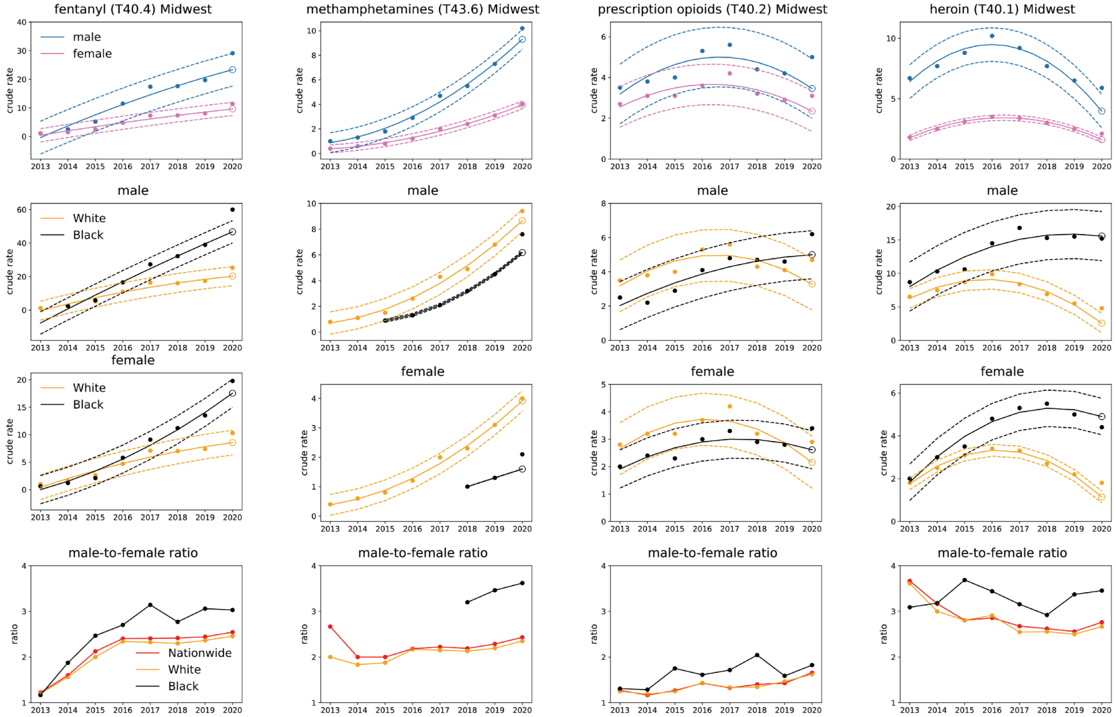

fentanyl (T40.4) Midwest
methamphetamines (T43.6) Midwest
prescription opioids (T40.2) Midwest
heroin (T40.1) Midwest
male
female
White
Black
male-to-female ratio
Nationwide
crude rate
ratio

C

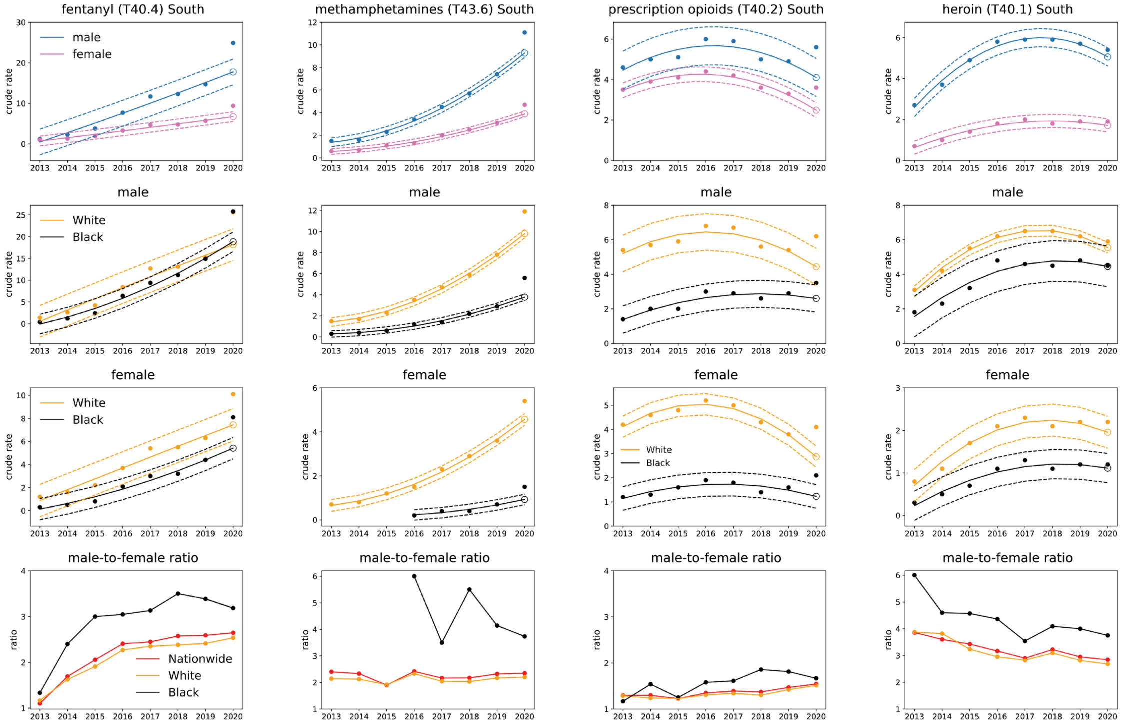

fentanyl (T40.4) South
methamphetamines (T43.6) South
prescription opioids (T40.2) South
heroin (T40.1) South
male
female
White
Black
male-to-female ratio
Nationwide
crude rate
ratio

D

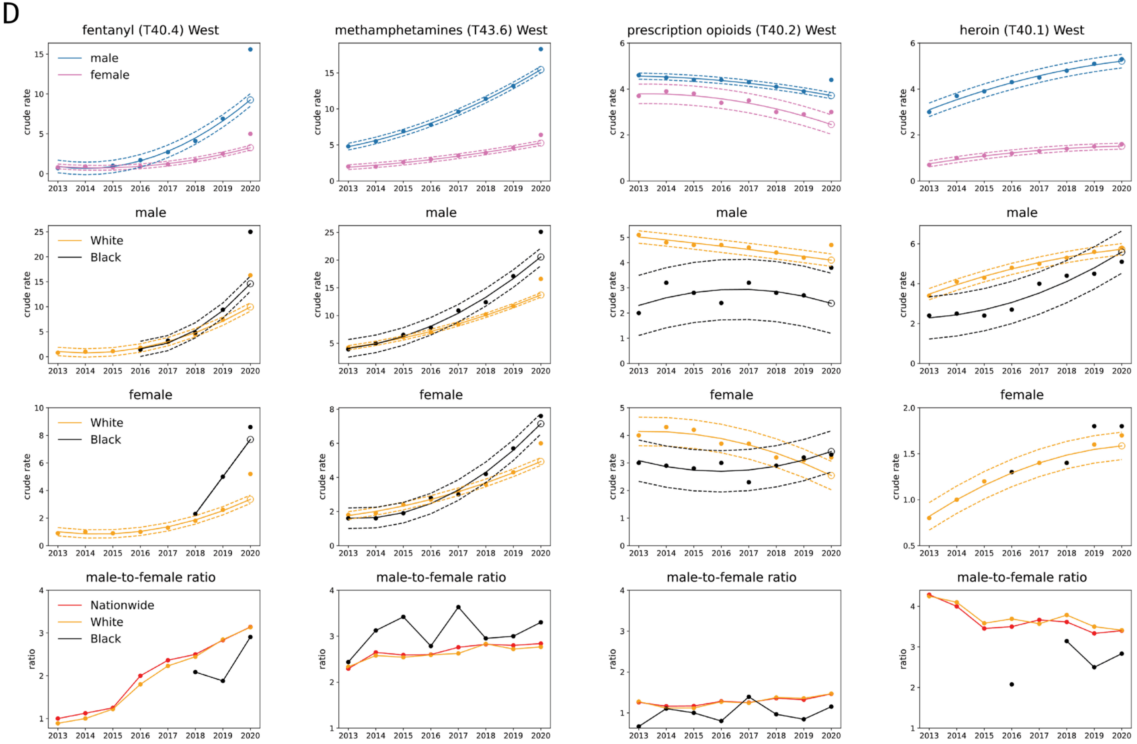

Figs A-D in S1 Text: Census region gender and race stratified crude rate trends for synthetic opioids (fentanyl, T40.4), psychostimulants with abuse potential (methamphetamines, T43.6), semi-synthetic opioids (prescription opioids, T43.6) and heroin (T40.1). Solid lines in the upper three rows represent the 2013-2019 quadratic regression; dotted lines correspond to values three times above and below the regression by three times the standard deviation calculated from 2013-2019 data; the open circle is the 2020 projection. Top row: results are stratified by gender. Second (third) row from top: results are stratified by race for males (females). Last row: Male-to-female ratio for all races. The four census regions are defined as: Northeast (Connecticut, Maine, Massachusetts, New Hampshire, New Jersey, New York, Pennsylvania, Rhode Island, Vermont), Midwest (Illinois, Indiana, Iowa, Kansas, Michigan, Minnesota, Missouri, Nebraska, North Dakota, Ohio, South Dakota, Wisconsin), South (Alabama, Delaware, District of Columbia, Florida, Georgia, Kentucky, Maryland, Mississippi, North Carolina, South Carolina, Tennessee, Virginia, West Virginia), West (Alaska, Arizona, California, Colorado, Hawaii, Idaho, Montana, Nevada, New Mexico, Oregon, Utah, Washington, Wyoming).
